# Intravascular brachytherapy vs. drug-coated balloons for in-stent restenosis in patients with diabetes

**DOI:** 10.3389/fcvm.2025.1634096

**Published:** 2026-01-12

**Authors:** Gal Sella, Gera Gandelman, Alex Blatt, Jacob George, Haitham Abu Khadija, Omar Ayyad, Devin Olek, Bin S. Teh, Yueh-Yun Lin, Anshuj Deva, Chloe Kharsa, Mangesh Kritya, Muhammad Faraz Anwaar, Joseph Elias, Elia El Hajj, Albert E. Raizner, Andrew Farach, Neal S. Kleiman, Alpesh Shah

**Affiliations:** 1 The Heart Center, Kaplan Medical Center, affiliated with the Hebrew University of Jerusalem, Rehovot, Israel; 2 Department of Cardiology, Houston Methodist DeBakey Heart and Vascular Center, Houston, TX, United States; 3 Department of Radiation Oncology, Houston Methodist Hospital, Houston, TX, United States; 4 Center for Health Data Science and Analytics, Houston Methodist, Houston, TX, United States

**Keywords:** brachytheraphy, drug coated balloon (DCB), in stent restenosis (ISR), PCI - percutaneous coronary intervention, revascualrization

## Abstract

**Background:**

Patients who have diabetes mellitus experience significantly higher rates of in-stent restenosis following percutaneous coronary intervention compared to the general population. The underlying pathophysiology of restenosis is exacerbated by diabetes-specific mechanisms including endothelial dysfunction, enhanced inflammatory response, and accelerated smooth muscle cell proliferation. While both intravascular brachytherapy (IVB) and drug-coated balloons (DCB) have been used to treat this condition, their comparative effectiveness in this high-risk population has never been evaluated in a long-term study.

**Objectives:**

To compare the efficacy and safety of IVB vs. DCB for the treatment of in-stent restenosis specifically in patients with diabetes.

**Methods:**

This dual-center study compared 2-year outcomes between patients with diabetes treated with IVB at Houston Methodist Hospital (USA) and DCB at Kaplan Medical Center (Israel). Propensity score matching was performed for age, sex, vessel size, and ejection fraction. Primary outcomes included all-cause mortality and target lesion failure (TLF).

**Results:**

DCB treatment was associated with shorter procedure times (58.2 ± 26.1 vs. 83.4 ± 37.2 min, *p* < 0.01) and reduced contrast use (121.5 ± 53.2 vs. 158.7 ± 73.5 mL, *p* = 0.03). In the propensity-matched cohort, MACE rates were similar (46.8% vs. 50.2%, *p* = 0.62). DCB treatment demonstrated significantly lower TLF rates compared to IVB (5.2% vs. 21.3%, *p* < 0.01) and reduced target vessel myocardial infarctions (3.9% vs. 15.6%, *p* = 0.01). Cardiac death rates were similar between groups (7.8% vs. 5.2%, *p* = 0.48). The mortality signal was particularly pronounced among patients with diabetes aged ≥65 years (HR 4.82, 95% CI: 1.05–22.17) and those with reduced ejection fraction (HR 3.15, 95% CI: 1.03–9.64), while the TLF benefit was consistent across most subgroups within the diabetic population.

**Conclusions:**

In this first-ever comparison with 2-year follow-up in patients with diabetes, DCB was associated with similar MACE rates and cardiac mortality rates compared to IVB but demonstrated significantly lower target lesion failure. These findings suggest that while DCB offers superior efficacy for ISR treatment in patients with diabetes, careful patient selection is crucial, particularly considering diabetes-related comorbidities that strongly influence overall survival.

## Introduction

Diabetes mellitus represents a major risk factor for coronary artery disease and is associated with accelerated atherosclerosis, more complex coronary lesions, and poorer outcomes following percutaneous coronary intervention (PCI) (1). Despite substantial advances in stent technology, patients with diabetes who have diabetes continue to experience significantly higher rates of in-stent restenosis (ISR) compared to non-diabetic counterparts, with reported incidences of up to 30%–40% for bare metal stents and 15%–20% for drug-eluting stents (DES) (2, 3). This stark disparity highlights the unique challenges posed by diabetes in the context of coronary interventions.

The pathophysiology of ISR in patients with diabetes is multifactorial and involves several diabetes-specific mechanisms that distinguish it from non-diabetic restenosis. Hyperglycemia induces endothelial dysfunction and promotes an exaggerated inflammatory response, while insulin resistance stimulates smooth muscle cell proliferation and migration. Furthermore, the diabetic milieu—characterized by increased oxidative stress, advanced glycation end products (AGEs), and altered cytokine profiles—creates a prothrombotic and proinflammatory environment that accelerates neointimal hyperplasia and extracellular matrix production. These mechanisms collectively contribute to the aggressive restenotic process observed in patients with diabetes and underscore the need for specialized treatment approaches (4, 5).

The management of ISR in patients with diabetes poses unique challenges that extend beyond the technical complexity of the lesion itself. Patients with diabetes typically present with diffuse, multivessel disease, smaller vessel calibers, and longer lesions compared to their non-diabetic counterparts. Moreover, diabetes-associated comorbidities such as renal impairment, peripheral vascular disease, and heart failure further complicate treatment planning and influence long-term outcomes (6). These factors necessitate careful consideration of treatment modalities that can effectively address the restenotic process while minimizing procedural risks and optimizing long-term outcomes.

Intravascular brachytherapy (IVB) emerged in the late 1990s as a promising treatment for ISR, with particular interest in its application to patients with diabetes. The mechanism of action involves the delivery of localized radiation to inhibit the exaggerated smooth muscle cell proliferation and subsequent neointimal formation that characterizes diabetic restenosis. Early trials of IVB, including subanalyses of patients with diabetes in the GAMMA-1 and BETA-CATH trials, showed particularly encouraging results in this high-risk population, with significant reductions in restenosis rates compared to conventional angioplasty techniques (7, 8). However, the adoption of IVB was limited by logistical challenges, including the need for specialized radiation safety protocols, dedicated equipment, and trained personnel, as well as concerns about late thrombosis and edge effects that might be particularly problematic in patients with diabetes.

The advent of drug-coated balloons (DCB) in the mid-2000s represented a potentially transformative approach for diabetic ISR treatment. DCBs allow local delivery of antiproliferative agents, most commonly paclitaxel, to the vessel wall, thereby addressing the excessive proliferative response characteristic of diabetic restenosis. This approach offers several theoretical advantages for patients with diabetes: logistic simplicity, homogeneous drug distribution, absence of additional stent layers (particularly beneficial in small diabetic vessels), and preservation of vessel geometry. Multiple randomized trials have demonstrated the efficacy of DCBs in treating restenosis, though dedicated studies in exclusively diabetic populations have been limited. Subgroup analyses from trials such as PACCOCATH-ISR and PEPCAD-DES have suggested particular benefit in patients with diabetes, but the evidence remains inconclusive (9).

The availability and adoption of these therapies varies significantly across regions due to regulatory and practical considerations. Drug-coated balloons received European authorization in 2009 but only gained FDA approval for coronary applications in the United States in 2024, leading to regional differences in real-world experience and institutional protocols (10). Conversely, while intravascular brachytherapy maintains a presence in selected U.S. institutions, particularly for recalcitrant ISR in high-risk patients, its use worldwide has largely declined due to the widespread adoption of DCBs. Despite these advances and regional variations in practice, the optimal treatment strategy for ISR in patients with diabetes remains debatable.

This ongoing clinical equipoise underscores the need for direct comparative studies between IVB and DCB in the treatment of ISR specifically in patients with diabetes, who represent a distinct and challenging patient population. To date, there are no such studies in the literature. Accordingly, we designed an international, retrospective cohort study comparing intravascular brachytherapy and drug-coated balloons for the treatment of in-stent restenosis in patients with diabetes by analyzing data from two major cardiovascular centers. The study combines data from patients with diabetes treated with drug-coated balloons at Kaplan Medical Center in Israel and those treated with intravascular brachytherapy at Houston Methodist Hospital in the United States.

## Methods

### Study design and population

This international, retrospective cohort study analyzed data of patients with diabetes at two major cardiovascular centers: Kaplan Medical Center (KMC) in Rehovot, Israel, and Houston Methodist Hospital (HMH) in Houston, Texas, USA. The study protocol was reviewed and approved by the institutional ethics committees at both centers (Helsinki Committee for Human Rights at KMC [KMC-0231-18] and the Institutional Review Board at HMH [PRO00038448]). Given the retrospective nature of the study, the requirement for informed consent was waived by both committees; all data were fully anonymized before analysis.

We included consecutive patients with diabetes who underwent treatment for in-stent restenosis with either DCB at KMC or IVB at HMH between January 2014 and December 2022. Diabetes was defined according to current clinical guidelines (11). For the DCB cohort at KMC, eligible patients with diabetes were those with coronary lesions treated with DCB according to operator discretion based on clinical and anatomical characteristics. The IVB cohort at HMH included patients with diabetes who received brachytherapy for ISR according to institutional protocols.

In the DCB group, the presence of type D, E, or F dissections according to the NHLBI classification system (12) or deterioration of TIMI flow to < grade II necessitated bailout stenting. These bailout-stented cases were included in the final analysis under the DCB group based on intention-to-treat principles.

### Procedural techniques

#### Drug-coated balloon protocol (KMC)

Procedures were performed via femoral or radial access using at least 6 French introducer sheaths, following ESC guidelines for myocardial revascularization. Lesion preparation was mandatory and consisted of predilatation with uncoated balloons using a balloon-to-vessel ratio of 0.8–1.0 at pressures exceeding nominal pressure. Special attention was given to adequate lesion preparation in patients with diabetes, who often present with more calcified and complex lesions. Second-generation paclitaxel-iopromide DCBs (SeQuent® Please, B. Braun, Melsungen, Germany or Pantera LUX, Biotronik, Berlin, Germany) were then deployed in the absence of flow-limiting dissection.

#### Intravascular brachytherapy protocol (HMH)

Procedures were performed according to the vendor recommended protocol. After successful balloon angioplasty of the restenotic lesion, beta radiation was delivered using a 6F guide catheter with the Beta-Cath™ 3.5F System (Novoste Corporation, Norcross, GA). The source train is comprised of Strontium/Ytrium-90 seeds that come in three lengths. To ensure complete lesion coverage and account for edge effects, the radiation source train length was selected to exceed the angioplasty segment by 10 mm on each end. Due to guide catheter placement issues or specific patient conditions, reduced margins were accepted in some cases. Radiation dosing followed a vessel size-dependent protocol, with all doses prescribed at 2 mm from the radioactive source center: vessels diameters ≤ 3.35 received 18.4 Gy, while vessel diameters > 3.35 received 23 Gy. Long lesions that required multiple contiguous dwells had an overlap segment of approximately 10 mm.

### Antithrombotic regimen

All patients with diabetes received periprocedural anticoagulation with either intravenous heparin (70 IU/kg or 5,000 IU) or bivalirudin (0.75 mg/kg bolus followed by 1.75 mg/kg/hr infusion). At Houston Methodist Hospital, bivalirudin was the preferred anticoagulant. Additional doses were administered as needed to maintain therapeutic anticoagulation. The post-procedure antithrombotic regimen included clopidogrel (75 mg/day following a 300–600 mg loading dose) for a minimum of three months and lifelong aspirin (75–100 mg/day). In patients with diabetes with acute coronary syndromes, dual antiplatelet therapy was extended to 12 months. Prasugrel or ticagrelor could be substituted for clopidogrel based on current guidelines and clinical considerations, with particular attention to the known higher platelet reactivity and reduced clopidogrel response in patients with diabetes. Preloading with any of these medications was permitted.

### Study endpoints and definitions

The primary endpoint was clinically driven target lesion revascularization (TLR) at two years. Secondary endpoints included two-year all-cause mortality and major adverse cardiac events (MACE), defined as a composite of TLR, cardiac death, cardiovascular hospitalizations, and definite vessel thrombosis. Myocardial infarction was defined according to the Fourth Universal Definition (13) and was classified as target vessel-related when occurring in the territory of the treated vessel. Definite acute/subacute vessel thrombosis was classified using the Academic Research Consortium criteria (14) Deaths of undetermined cause were analyzed as cardiac in origin, following standard cardiovascular trial methodology; however, this conservative approach may potentially overestimate cardiac mortality, particularly in diabetic populations with multiple competing causes of death.

### Follow-up and data collection

Data collection was conducted independently at each participating center through established registry protocols. Both institutions maintained comprehensive databases that captured detailed patient information at the time of intervention, including baseline demographics, cardiovascular risk factors, clinical presentation, previous cardiac history, and complete procedural characteristics.

Post-procedure follow-up was conducted through a combination of clinical visits and medical record review. Repeat coronary angiography was performed when clinically indicated, such as in cases of recurrent symptoms or objective evidence of ischemia. The follow-up data collection process included documentation of all major adverse cardiac events, with particular attention to target lesion revascularization procedures, myocardial infarctions, and cardiovascular-related hospitalizations.

### Statistical analysis

For continuous variables, the mean and standard deviation (SD) were presented for the descriptive analysis. Two-sample independent *T*-tests were conducted if the variables were normally distributed. If the variables were not normally distributed, the *p*-values were derived from the Wilcoxon rank-sum test.

For categorical variables, the count and percentage (%) were presented for the descriptive analysis. The Chi-square test was conducted if the expected value was ≥5, where one of the expected values in the contingency table between the categorical variable and Treatment (Intravascular Brachytherapy, IVB, and Drug-coated balloon, DCB). Otherwise, Fisher's exact test was conducted.

A 1:1 propensity score matching using logistic regression was performed to control for the effect of potential covariates. Nearest-neighbor matching without replacement with a caliper of 0.05 was conducted to ensure comparability between IVB and DCB patients. Age, sex, vessel size ≥2.75 mm, and ejection fraction (EF) < 50% were balanced based on their clinical significance as established prognostic factors in coronary interventions. These four variables were selected based on their proven independent predictive value for ISR treatment outcomes in prior literature; sample size constraints (*n* = 174) limited matching to this focused set to maintain adequate statistical power while optimizing covariate balance. The standardized mean difference (SMD) was calculated as the balance diagnostics after propensity score matching, with SMD <0.2 considered acceptable balance. (Supplementary Figure A1).

The probabilities of survival for all-cause mortality and target lesion failure (TLF) were estimated by the Kaplan–Meier method and are presented as Kaplan–Meier curves. All-cause death was analyzed as a competing risk for the probability estimation of TLF.

The hazard ratio was estimated using the Cox proportional hazards regression, with subgroup analysis for age ≥ 65, sex, body surface area ≥ 1.8, EF < 50, chronic kidney disease and vessel size ≥ 2.75.

The significance level was set at *α* = 0.10. All quantitative analyses were conducted using Stata/MP 17.0 and Python 3.12.7.

## Results

A total of 174 patients with diabetes were included in the initial analysis (IVB: *n* = 110; DCB: *n* = 64). After propensity score matching, 55 pairs were analyzed.

### Baseline characteristics

Prior to matching, the diabetic IVB group had higher rates of hypertension (97.3% vs. 85.9%, *p* = 0.01) and hyperlipidemia (93.6% vs. 81.3%, *p* = 0.01). EF < 50% was more prevalent in the diabetic DCB group (54.7% vs. 31.8%, *p* < 0.01). Body surface area was higher in the diabetic IVB group (2.04 ± 0.28 vs. 1.89 ± 0.18, *p* < 0.01). The DCB group was slightly older (67.8 ± 9.9 vs. 64.6 ± 9.8 years, *p* = 0.04) (Table 1).

**Table 1:** Demographic characteristics between the IVB and DCB diabetes patient pre- and post-matching.

Characteristic	Pre-matching				Post-matching			
	IVB (*n* = 110)	DCB (*n* = 64)	SMD	*p*-value	IVB (*n* = 55)	DCB (*n* = 55)	SMD	*p*-value
Age, year	64.59 (9.75)	67.75 (9.93)	−0.32	0.04	65.40 (10.71)	67.55 (9.93)	−0.21	0.28
Sex			0.05	0.73			0.18	0.36
Female	25 (22.73%)	16 (25.00%)			10 (18.18%)	14 (25.45%)		
Male	85 (77.27%)	48 (75.00%)			45 (81.82%)	41 (74.55%)		
Body surface area	2.04 (0.28)	1.89 (0.18)	0.68	<0.01	2.05 (0.28)	1.89 (0.17)	0.68	<0.01
Comorbidities								
EF < 50 (ref: EF ≥ 50)	35 (31.82%)	35 (54.69%)	−0.47	<0.01	31 (56.36%)	27 (49.09%)	0.14	0.45
CKD	35 (31.82%)	23 (35.94%)	−0.09	0.58	17 (30.91%)	19 (34.55%)	−0.08	0.68
COPD	15 (13.64%)	4 (6.25%)	0.25	0.13	7 (12.73%)	4 (7.27%)	0.18	0.34
Hypertension	107 (97.27%)	55 (85.94%)	0.41	0.01	53 (96.36%)	47 (85.45%)	0.38	0.47
Hyperlipidemia	103 (93.64%)	52 (81.25%)	0.38	0.01	51 (92.73%)	44 (80.00%)	0.37	0.05

Post-matching, balance improved for age (67.6 ± 9.9 vs. 65.4 ± 10.7 years, *p* = 0.28), sex (*p* = 0.36), EF < 50% (49.1% vs. 56.4%, *p* = 0.45), and COPD (7.3% vs. 12.7%, *p* = 0.34). Hypertension remained more prevalent in the diabetic IVB group (96.4% vs. 85.5%, *p* = 0.47), as did hyperlipidemia (92.7% vs. 80.0%, *p* = 0.05) and body surface area (2.05 ± 0.28 vs. 1.89 ± 0.17, *p* < 0.01) (Table 1).

### Procedural characteristics

The diabetic IVB group had larger vessel diameters (3.48 ± 0.59 vs. 3.05 ± 0.59 mm, *p* < 0.01) but lesions had comparable length (27.81 ± 18.44 vs. 23.45 ± 6.24 mm, *p* = 0.32). IVB procedures were substantially longer (81.81 ± 37.19 vs. 58.77 ± 24.68 min, *p* < 0.01) with more contrast use (151.06 ± 59.76 vs. 101.71 ± 46.80 mL, *p* < 0.01). Radiation dose-area product (DAP) was lower in the IVB group (14,856.29 ± 13,739.81 vs. 51,810.42 ± 32,223.00 mGy/cm^2^, *p* < 0.01) (Table 2).

**Table 2:** Procedure characteristics between the IVB and DCB diabetes patient pre- and post-matching.

Characteristic	Pre-matching				Post-matching			
	IVB (*n* = 110)	DCB (*n* = 64)	SMD	*p*-value	IVB (*n* = 55)	DCB (*n* = 55)	SMD	*p*-value
Procedure length, minutes	81.81 (37.19)	58.77 (24.68)	0.73	<0.01	86.33 (36.88)	58.15 (25.88)	0.88	<0.01
Radiation Dose, mGy/cm^2^	14,856.29 (13,739.81)	51,810.42 (32,223.00)	−1.49	<0.01	17,434.33 (16,364.47)	47,529.15 (31,062.25)	−1.21	<0.01
Contrast volume	151.06 (59.76)	101.71 (46.80)	0.92	<0.01	157.83 (63.65)	103.77 (48.48)	0.96	<0.01
Vessel size, mm	3.48 (0.59)	3.05 (0.59)	0.73	<0.01	3.43 (0.68)	3.12 (0.59)	0.49	0.01
Vessel size <2.75 mm	9 (8.33%)	19 (30.65%)	0.58	<0.01	9 (16.36%)	13 (23.64%)	0.18	0.34
Lesion length (mm)	27.81 (18.44)	23.45 (6.24)	0.32	0.65	28.45 (19.23)	22.89 (5.82)	0.39	0.84
Artery			0.03	0.78			0.01	0.87
LAD	37 (33.64%)	22 (34.38%)			21 (38.18%)	18 (32.73%)		
LCX	28 (25.45%)	20 (31.25%)			14 (25.45%)	17 (30.91%)		
RCA	30 (27.27%)	14 (21.88%)			10 (18.18%)	13 (23.64%)		
RI	5 (4.55%)	1 (1.56%)			3 (5.45%)	1 (1.82%)		
LMCA	6 (5.45%)	3 (4.69%)			4 (7.27%)	3 (5.45%)		
Balloon type			0.11	<0.01			0.19	0.02
Semi-complaint balloon	11 (10.28%)	26 (40.63%)			10 (18.18%)	24 (43.64%)		
Non-compliant balloon	69 (64.49%)	31 (48.44%)			31 (56.36%)	25 (45.45%)		
Scoring balloon	5 (4.67%)	1 (1.56%)			2 (3.64%)	1 (1.82%)		
Cutting balloon	19 (17.76%)	6 (9.38%)			9 (16.36%)	5 (9.09%)		
Bailout stenting	–	4 (6.25%)	–	–	–	2 (3.64%)	–	–
Number of DCBs used								
1	–	57 (91.94%)	–	–	–	51 (92.73%)	–	–
2	–	4 (6.45%)	–	–	–	3 (5.45%)	–	–
3	–	1 (1.61%)	–	–	–	1 (1.82%)	–	–

Vessel size <2.75 mm was more common in the DCB group (30.7% vs. 8.3%, *p* < 0.01). The most common target vessel in both diabetic groups was the left anterior descending artery (IVB: 33.6% vs. DCB: 34.4%). The distribution of treated vessels was similar between groups (*p* = 0.78). Non-compliant balloons were most common in the IVB group (64.5%), while semi-compliant balloons were more common in the DCB group (40.6%, *p* < 0.01). Bailout stenting was required in 6.3% of DCB cases (Table 2).

After matching, some procedural differences remained significant. The IVB group continued to have longer procedures (86.33 ± 36.88 vs. 58.15 ± 25.88 min, *p* < 0.01), greater contrast use (157.83 ± 63.65 vs. 103.77 ± 48.48 mL, *p* < 0.01), larger vessel diameters (3.43 ± 0.68 vs. 3.12 ± 0.59 mm, *p* = 0.01). lesion length was comparable (28.45 ± 19.23 vs. 22.89 ± 5.82 mm, *p* = 0.84) as well as small vessels (16.36% vs. 23.64%, *p* = 0.34) (Table 2).

### Clinical outcomes

In the pre-matched cohort, the DCB group had significantly fewer target lesion revascularizations (6.3% vs. 23.6%, *p* < 0.01) compared to the IVB group. MACE rates were similar (48.4% vs. 50.9%, *p* = 0.75). Myocardial infarction rates were numerically lower in the DCB group but did not reach statistical significance (4.7% vs. 11.8%, *p* = 0.12). All-cause mortality was significantly higher with DCB (23.4% vs. 11.8%, *p* = 0.04), while cardiac death rates were similar (7.8% vs. 4.6%, *p* = 0.50) (Table 3).

**Table 3:** Outcome characteristics between the IVB and DCB diabetes patient pre- and post-matching.

Characteristic	Pre-matching				Post-matching			
	IVB (*n* = 110)	DCB (*n* = 64)	SMD	*p*-value	IVB (*n* = 55)	DCB (*n* = 55)	SMD	*p*-value
Major adverse cardiac events	56 (50.91%)	31 (48.44%)	0.05	0.75	27 (49.09%)	26 (47.27%)	0.04	0.85
Target lesion revascularization, TLR	26 (23.64%)	4 (6.25%)	0.50	<0.01	12 (21.82%)	4 (7.27%)	0.42	0.03
Thrombosis	3 (2.73%)	0 (0.00%)	0.24	0.30	3 (5.45%)	0 (0.00%)	0.34	0.24
Bleeding	4 (3.64%)	0 (0.00%)	0.27	0.30	2 (3.64%)	0 (0.00%)	0.27	0.50
Cardiac hospitalization	43 (39.09%)	25 (39.06%)	0.00	>0.99	22 (40.00%)	20 (36.36%)	0.07	0.70
Myocardial infarctions	13 (11.82%)	3 (4.69%)	0.26	0.12	7 (12.73%)	3 (5.45%)	0.25	0.19
All-cause mortality	13 (11.82%)	15 (23.44%)	−0.31	0.04	6 (10.91%)	12 (21.82%)	−0.30	0.12
Cardiac death	5 (4.55%)	5 (7.81%)	−0.14	0.50	4 (7.27%)	5 (9.09%)	−0.07	>0.99

In the matched diabetic cohort, the DCB group maintained significantly lower target lesion revascularization rates (7.3% vs. 21.8%, *p* = 0.03). MACE rates remained similar (47.3% vs. 49.1%, *p* = 0.85). Myocardial infarctions were numerically lower with DCB but not statistically significant (5.5% vs. 12.7%, *p* = 0.19). All-cause mortality continued to be numerically higher with DCB, though not reaching statistical significance in the matched cohort (21.8% vs. 10.9%, *p* = 0.12). Cardiac death rates remained similar (9.1% vs. 7.3%, *p* > 0.99). No thrombosis or bleeding events were observed in the DCB group, compared to 5.5% thrombosis and 3.6% bleeding in the IVB group, though these differences were not statistically significant (Table 3).

### Survival analysis

**Figure 1:**
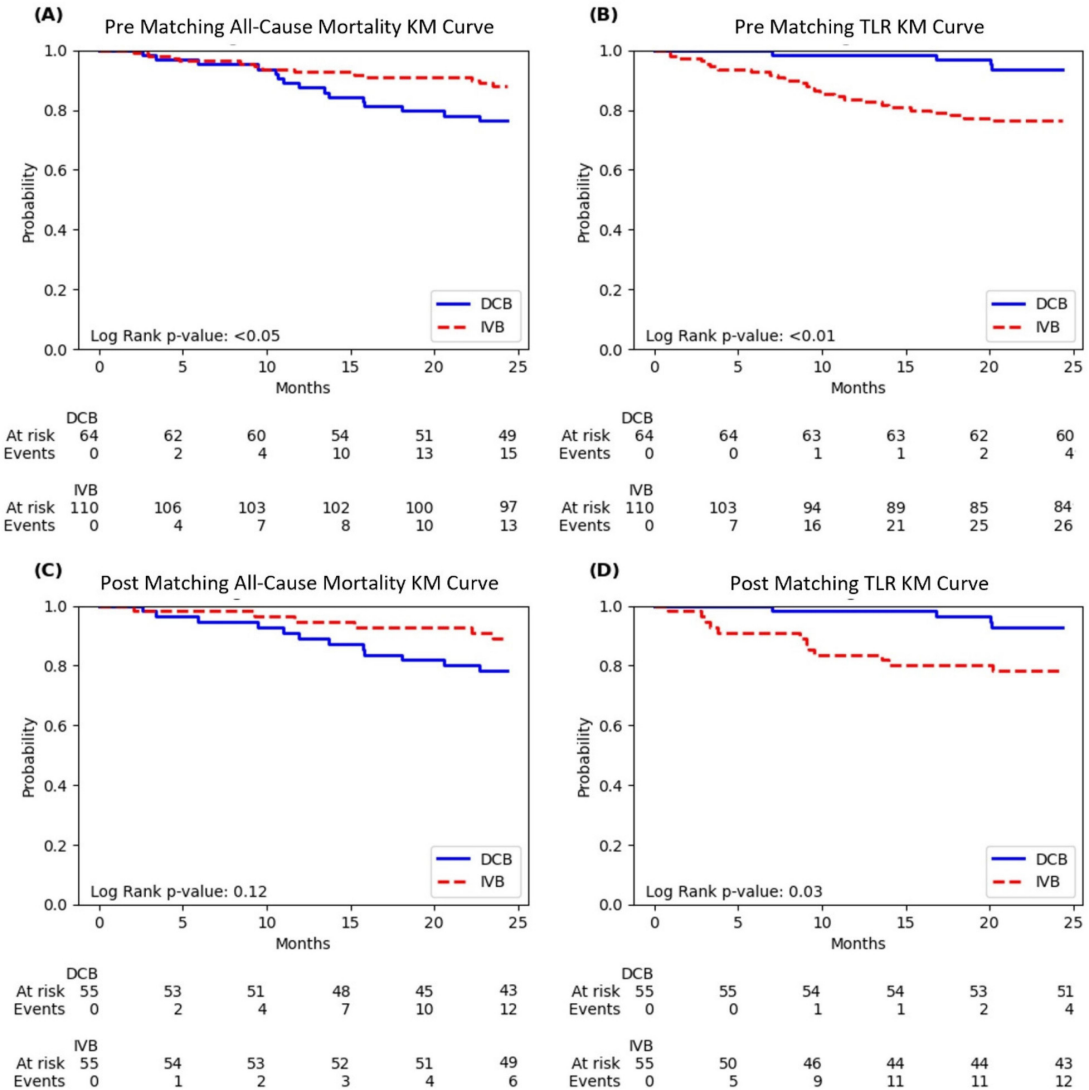
Kaplan–Meier curves of all-cause mortality and target lesion revascularization (TLR). **(A)** Pre-matching all-cause mortality; **(B)** Pre-matching TLR; **(C)** Post-matching all-cause mortality; **(D)** Post-matching TLR. Curves compare drug-coated balloon (DCB) and intravascular brachytherapy (IVB) treatments. Log-rank p-values are shown in each panel, with numbers at risk and events displayed below each curve.

Kaplan–Meier curves for all-cause mortality and target lesion revascularization (TLR) before and after propensity score matching showed distinctive patterns in the diabetic population (Figure 1).

The unmatched all-cause mortality curves showed a clear separation between the treatment groups, with DCB having significantly lower survival probability than IVB throughout the 24-month follow-up period (log-rank *p* < 0.05). At 24 months, the diabetic DCB group had 15 events (23.4%) compared to 13 events (11.8%) in the IVB group (Figure 1).

For unmatched TLR, the curves demonstrated pronounced and immediate separation in the opposite direction, with the DCB group maintaining significantly higher TLR-free survival throughout follow-up (log-rank *p* < 0.01). By 24 months, the diabetic DCB group had experienced only 4 TLR events (6.3%) compared to 26 events (23.6%) in the IVB group (Figure 1).

After propensity score matching, the all-cause mortality separation remained visible, though it became statistically non-significant (log-rank *p* = 0.12). At 24 months, the matched diabetic DCB group had 12 events (21.8%) compared to 6 events (10.9%) in the matched IVB group (Figure 1).

The post-matching TLR curves continued to show significant benefit with DCB (log-rank *p* = 0.03), with only 4 events (7.3%) in the diabetic DCB group vs. 12 events (21.8%) in the IVB group at 24 months. The separation of these curves was evident early and maintained throughout the follow-up period (Figure 1).

### Subgroup analysis

**Figure 2:**
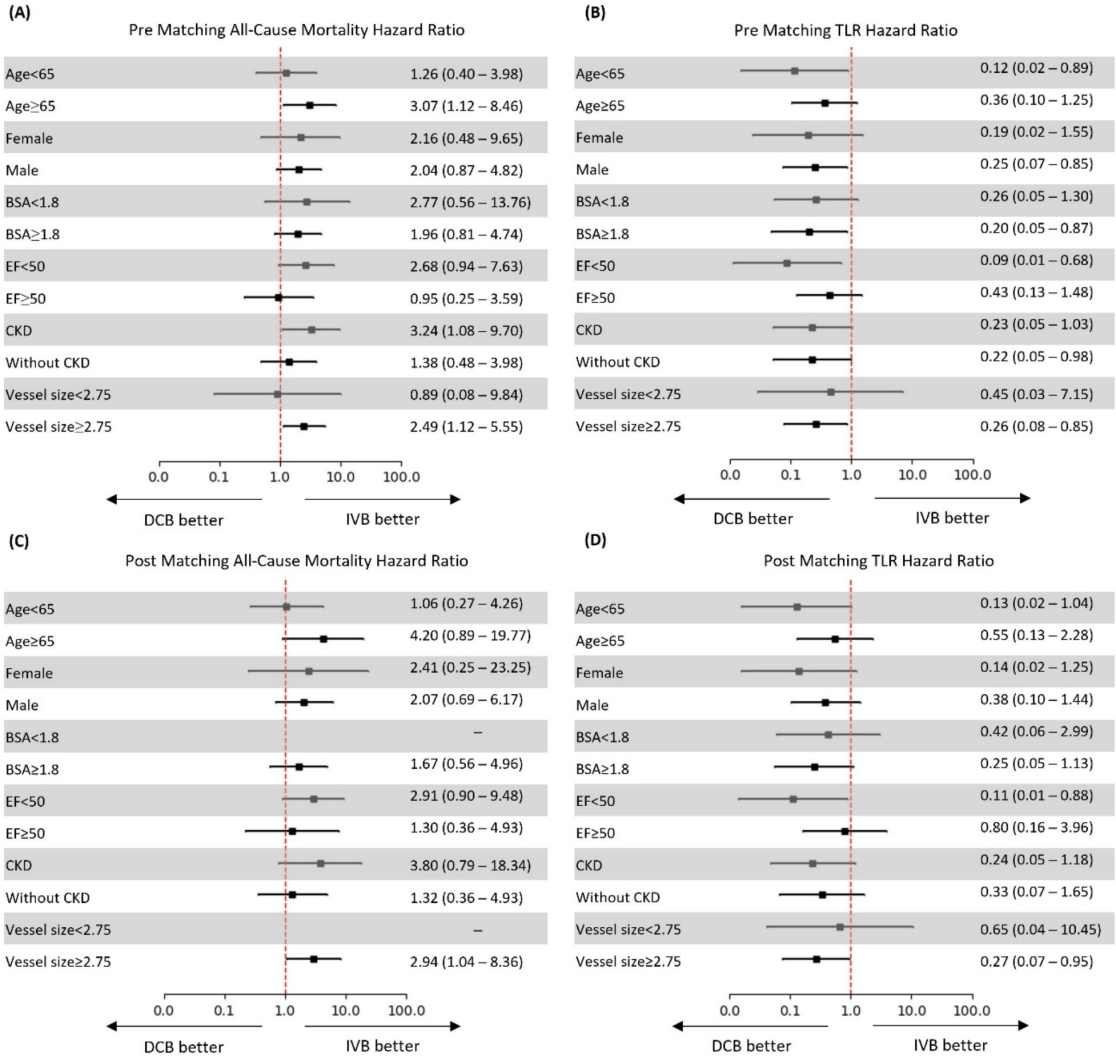
Hazard ratios for all-cause mortality and target lesion revascularization (TLR) before and after propensity score matching. **(A)** Pre-matching all-cause mortality; **(B)** Pre-matching TLR; **(C)** Post-matching all-cause mortality; **(D)** Post-matching TLR. Hazard ratios with 95% confidence intervals are shown for predefined subgroups. Directional arrows indicate “DCB better” and “IVB better”.

Subgroup analysis of hazard ratios for both mortality and TLR revealed several important patterns in the diabetic population (Figure 2). For all-cause mortality pre-matching, DCB treatment was associated with increased risk across most subgroups, with particularly pronounced effects in patients aged ≥65 years (HR 3.07, 95% CI: 1.12–8.46), those with CKD (HR 3.24, 95% CI: 1.08–9.70), and patients with vessel size ≥2.75 mm (HR 2.49, 95% CI: 1.12–5.55). Interestingly, patients with EF < 50% showed no significant increase in mortality risk (HR 0.95, 95% CI: 0.25–3.59) and those with vessel size <2.75 mm had a numerically lower risk with DCB, though not statistically significant (HR 0.89, 95% CI: 0.08–9.84) (Figure 2).

In the post-matching analysis, the mortality risk associated with DCB was amplified in several subgroups, notably patients aged ≥65 years (HR 4.20, 95% CI: 0.89–19.77), those with CKD (HR 3.80, 95% CI: 0.79–18.34), and patients with vessel size ≥2.75 mm (HR 2.94, 95% CI: 1.04–8.36). The last subgroup demonstrated a statistically significant increase in mortality with DCB. Patients with EF < 50% showed a trend toward higher mortality with DCB (HR 1.30, 95% CI: 0.36–4.93), while those with EF ≥ 50% had a stronger signal (HR 2.91, 95% CI: 0.50–9.48).

DCB demonstrated a consistent protective effect against TLR across most diabetic subgroups both pre- and post-matching. Pre-matching, the strongest benefit was observed in patients with EF < 50% (HR 0.09, 95% CI: 0.01–0.68), those aged <65 years (HR 0.12, 95% CI: 0.02–0.89), and female patients (HR 0.19, 95% CI: 0.02–1.55). The protective effect was statistically significant for patients without CKD (HR 0.22, 95% CI: 0.05–0.98) and male patients (HR 0.25, 95% CI: 0.07–0.85) (Figure 2).

Post-matching analysis confirmed this protective effect, with the most pronounced benefit seen in patients with EF < 50% (HR 0.11, 95% CI: 0.01–0.88), those aged <65 years (HR 0.13, 95% CI: 0.02–1.04), and female patients (HR 0.14, 95% CI: 0.02–1.25). Notably, the protective effect of DCB against TLR remained statistically significant for patients with vessel size ≥2.75 mm (HR 0.27, 95% CI: 0.07–0.95), while the benefit was less pronounced in patients with smaller vessels <2.75 mm (HR 0.65, 95% CI: 0.04–10.45), suggesting that vessel size may significantly influence the efficacy of DCB treatment in patients with diabetes (Figure 2).

## Discussion

This international, dual-center study provides the first direct comparison of intravascular brachytherapy (IVB) and drug-coated balloons (DCB) for treating in-stent restenosis (ISR) specifically in patients with diabetes. As diabetes is a well-established risk factor for ISR development and recurrence, understanding optimal treatment strategies for this high-risk population is particularly important.

Our data demonstrate distinct differences in both procedural characteristics and clinical outcomes between these two treatment modalities in patients with diabetes. Procedurally, DCB offered substantial advantages, with significantly shorter procedure times (58.8 ± 24.7 vs. 81.8 ± 37.2 min, *p* < 0.01) and markedly reduced contrast volumes (101.7 ± 46.8 vs. 151.1 ± 59.8 mL, *p* < 0.01). These procedural benefits are especially relevant for patients with diabetes, who frequently present with concurrent nephropathy and are at higher risk for contrast-induced kidney injury (15).

The clinical outcomes at two years revealed a complex efficacy-safety balance that challenges conventional treatment paradigms for patients with diabetes with ISR. Despite similar overall MACE rates between treatment groups (47.3% for DCB vs. 49.1% for IVB, *p* = 0.85), the individual components driving these events differed substantially. DCB treatment was associated with significantly lower target lesion revascularization (TLR) rates (7.3% vs. 21.8%, *p* = 0.03), representing a 67% relative risk reduction. This finding is consistent with previous studies such as the PEPCAD-DES and PACCOCATH-ISR trials, which showed pronounced antirestenotic effects of DCB in diabetic subgroups (9).

Conversely, our findings revealed a concerning signal regarding all-cause mortality. Before propensity matching, DCB treatment was associated with significantly higher all-cause mortality (23.4% vs. 11.8%, *p* = 0.04). After matching, although no longer statistically significant, this Importantly, cardiac mortality rates were similar between groups (9.1% vs. 7.3%, *p* > 0.99), suggesting that the excess mortality with DCB was driven by non-cardiac causes. This pattern, combined with the international, dual-center design of our study, raises the possibility that the mortality difference may reflect unmeasured confounders related to differences in patient populations between institutions rather than a direct effect of DCB treatment. As DCB in usual clinical practice is often used for patients with higher comorbidity burdens, residual confounding not fully addressed by propensity score matching may explain this signal (16).

Our subgroup analysis provided critical insights into the differential effects of these treatments. The mortality signal with DCB was most pronounced in specific subgroups: patients aged ≥65 years (HR 4.20, 95% CI: 0.89–19.77), those with CKD (HR 3.80, 95% CI: 0.79–18.34), and patients with larger vessel sizes ≥2.75 mm (HR 2.94, 95% CI: 1.04–8.36) - with the last subgroup demonstrating statistical significance.

The protective effect of DCB against TLR was most substantial in patients with reduced ejection fraction (HR 0.11, 95% CI: 0.01–0.88), younger patients <65 years (HR 0.13, 95% CI: 0.02–1.04), and females (HR 0.14, 95% CI: 0.02–1.25). The vessel size-dependent effect was particularly noteworthy - DCB showed significant benefit in vessels ≥2.75 mm (HR 0.27, 95% CI: 0.07–0.95) but less pronounced effect in smaller vessels (HR 0.65, 95% CI: 0.04–10.45). The diminished efficacy in smaller vessels may reflect challenges in delivering adequate drug concentrations in the complex, tortuous, and often calcified small vessels characteristic of patients with diabetes.

The procedural characteristics we observed may influence clinical outcomes. The IVB group had more thrombosis (5.5% vs. 0%) and bleeding events (3.6% vs. 0%), although these differences did not reach statistical significance. This observation aligns with previous studies suggesting that radiation therapy may delay endothelialization and predispose to late thrombotic events, particularly in patients with diabetes who already exhibit prothrombotic tendencies (17, 18).

Integrating our findings with existing literature suggests that treatment selection for patients with diabetes with ISR should be individualized based on patient and lesion characteristics. For patients with diabetes <65 years with reduced ejection fraction and adequate vessel size, DCB may represent the preferred strategy due to its superior efficacy in preventing recurrent restenosis. Conversely, in older patients with preserved ejection fraction, especially those with CKD or larger vessels, the mortality signal associated with DCB warrants careful consideration, and IVB might be advantageous despite its higher TLR rates.

## Limitations

Several important limitations of our study must be acknowledged. First, as a retrospective, observational study, selection bias cannot be entirely eliminated despite propensity score matching. The dual-center, international design introduces potential confounders related to different healthcare systems, practice patterns, and post-procedure medical regimens. These differences are particularly relevant when comparing patients with diabetes, whose management may vary significantly between institutions.

Second, the relatively small sample size (110 patients with diabetes in the IVB group and 64 in the DCB group) limits statistical power, especially for subgroup analyses. Despite propensity matching, standardized mean differences remained >0.2 for several baseline characteristics, including body surface area (SMD = 0.68), indicating incomplete balance between treatment groups.

Third, we lack detailed information about diabetes-specific factors that might influence outcomes, such as glycemic control, diabetes duration, insulin requirements, and diabetic microvascular complications. Additionally, we did not take into account for differences in antithrombotic regimens, which are particularly relevant in patients with diabetes who often require more intensive and prolonged antiplatelet therapy due to their prothrombotic state.

Finally, the absence of routine angiographic follow-up prevents distinction between clinical and subclinical restenosis. This is especially important in patients with diabeteswho may present with atypical symptoms or silent ischemia, potentially underestimating the true restenosis rate. Additionally, information on the type of initially implanted stent (bare-metal vs. drug-eluting) and the duration from original stent implantation to ISR presentation was not systematically available; these factors may influence ISR pathophysiology and treatment response. Recent evidence has also highlighted the importance of lesion characteristics such as unstented restenotic segment length in predicting outcomes after DCB treatment for ISR, which we did not specifically analyze. Additionally, we did not assess the angiographic pattern of in-stent restenosis (focal vs. diffuse), which recent evidence suggests significantly influences treatment outcomes; drug-eluting balloons may be more effective for focal ISR lesions, while diffuse ISR patterns may require repeated stenting with drug-eluting stents (19).patients with diabetes, a population frequently underrepresented in cardiovascular trials.

## Conclusions

This first comparison between IVB and DCB for treating in-stent restenosis in patients with diabetes reveals a nuanced risk-benefit profile that may inform clinical decision-making and propense further controlled studies. DCB demonstrated superior efficacy with significantly lower target lesion revascularization rates (7.3% vs. 21.8%, *p* = 0.03) and practical advantages including shorter procedure times and reduced contrast use. However, the signal for higher all-cause mortality with DCB (21.8% vs. 10.9%, *p* = 0.12), which was statistically significant before matching (23.4% vs. 11.8%, *p* = 0.04), warrants careful consideration, particularly since cardiac mortality rates remained similar between groups.

Our detailed subgroup analysis identified important patient factors that modify treatment outcomes. The mortality risk associated with DCB was significantly elevated in patients with larger vessels ≥2.75 mm (HR 2.94, 95% CI: 1.04–8.36) and showed strong trends in older patients ≥65 years (HR 4.20, 95% CI: 0.89–19.77) and those with CKD (HR 3.80, 95% CI: 0.79–18.34). Conversely, the protective effect against TLR was most pronounced in patients with reduced ejection fraction (HR 0.11, 95% CI: 0.01–0.88), younger diabetics (HR 0.13, 95% CI: 0.02–1.04), and females (HR 0.14, 95% CI: 0.02–1.25).

These findings suggest that treatment selection should be guided by specific patient characteristics. For younger patients with diabetes (<65 years) with reduced ejection fraction, DCB appears to offer substantial benefits with lower risks. For older patients (≥65 years), especially those with CKD or larger vessels, a more cautious approach to DCB use is warranted, with IVB potentially offering a more favorable risk-benefit profile despite higher TLR rates. Those observations still needs validation from larger, prospective trials.

Future research should focus on validating these subgroup findings in larger cohorts, identifying specific diabetes-related factors that modify treatment outcomes, and investigating whether intensified antithrombotic regimens might mitigate procedure-specific risks. These findings underscore the complexity of treating coronary disease in diabetes and highlight the need for a personalized approach to ISR management in this challenging patient population.

## Data Availability

The raw data supporting the conclusions of this article will be made available by the authors, without undue reservation.

## Supplementary material

Supplementary Figure A1 Balance diagnostic pre- and post- matching by characteristics.

## References

[1] Arnold SV Bhatt DL Barsness GW Beatty AL Deedwania PC Inzucchi SE Clinical management of stable coronary artery disease in patients with type 2 diabetes mellitus: a scientific statement from the American Heart Association. Circulation. (2020) 141(19):e779–806. 10.1161/CIR.0000000000000766 32279539 PMC12204403

[2] Qin S-Y Zhou Y Jiang H-X Hu B-L Tao L Xie M-z. The association of diabetes mellitus with clinical outcomes after coronary stenting: a meta-analysis. PLoS One. (2013) 8:e72710. 10.1371/journal.pone.0072710 24066025 PMC3774683

[3] Bangalore S Bates ER Beckie TM Bischoff JM Bittl JA Cohen MG 2021 ACC/AHA/SCAT guideline for coronary artery revascularization. J Am Coll Cardiol. (2022) 79(2):E21–129. 10.1016/j.jacc.2021.09.006 34895950

[4] Aronson D Bloomgarden Z Rayfield EJ. Potential mechanisms promoting restenosis in diabetic patients. J Am Coll Cardiol. (1996) 27:528–35. 10.1016/0735-1097(95)00496-3 8606261

[5] Yan SF Ramasamy R Naka Y Schmidt AM. Glycation, inflammation, and RAGE: a scaffold for the macrovascular complications of diabetes and beyond. Circ Res. (2003) 93:1159–69. 10.1161/01.RES.0000103862.26506.3D 14670831

[6] Jakubiak GK Pawlas N Cieślar G Stanek A. Pathogenesis and clinical significance of in-stent restenosis in patients with diabetes. Int J Environ Res Public Health. (2021) 18:11970. 10.3390/ijerph182211970 34831726 PMC8617716

[7] Moses JW Moussa I Leon MB Teirstein PS Fish RD Ellis SG Effect of catheter-based iridium-192 gamma brachytherapy on the added risk of restenosis from diabetes mellitus after intervention for in-stent restenosis (subanalysis of the GAMMA I randomized trial). Am J Cardiol. (2002) 90(3):243–7. 10.1016/S0002-9149(02)02462-1 12127611

[8] Suntharalingam M Laskey WK Tantibhedhyangkul W Lansky A Teirstein P Bass T Vascular brachytherapy using a beta emitter source in diabetic patients with in-stent restenosis: angiographic and clinical outcomes. Int J Radiat Oncol Biol Phys. (2003) 57(2):536–42. 10.1016/S0360-3016(03)00537-6 12957267

[9] Verdoia M Nardin M Rognoni A Cortese B. Drug-coated balloons in high-risk patients and diabetes mellitus: a meta-analysis of 10 studies. Catheter Cardiovasc Interv. (2024) 104:1423–33. 10.1002/ccd.31257 39465638

[10] FDA. AGENT Paclitaxel-Coated Balloon Catheter – P230035. (2024).

[11] Marx N Federici M Schuett K Mueller-Wieland D Ajjan RA Antunes MJ 2023 ESC guidelines for the management of cardiovascular disease in patients with diabetes: developed by the task force on the management of cardiovascular disease in patients with diabetes of the European Society of Cardiology (ESC). Eur Heart J. (2023) 44(39):4043–140. 10.1093/eurheartj/ehad192 37622663

[12] Huber MS Mooney JF Madison J Mooney MR. Use of a morphologic classification to predict clinical outcome after dissection from coronary angioplasty. Am J Cardiol. (1991) 68:467–71. 10.1016/0002-9149(91)90780-O 1872273

[13] Thygesen K Alpert JS Jaffe AS Chaitman BR Bax JJ Morrow DA Fourth universal definition of myocardial infarction (2018). J Am Coll Cardiol. (2018) 72(18):2231–64. 10.1016/j.jacc.2018.08.1038 30153967

[14] Garcia HG McFadden EP Farb A Mehran R Stone GW Spertus J Standardized end point definitions for coronary intervention trials. Eur Heart J. (2018) 39(23):2192–207. 10.1093/eurheartj/ehy223 29897428

[15] Lee CC Chan YL Wong YC Ng CJ Chang CH Hung CC Contrast-enhanced CT and acute kidney injury: risk stratification by diabetic status and kidney function. Radiology. (2023) 307(5):e222321. 10.1148/radiol.222321 37278631

[16] Jeger RV Eccleshall S Wan Ahmad WA Ge J Poerner TC Shin ES Drug-coated balloons for coronary artery disease: third report of the international DCB consensus group. Card Interv. (2020) 13(12):1391–402. 10.1016/j.jcin.2020.02.043 32473887

[17] Sabaté M Pimentel G Prieto C Corral JM Bañuelos C Angiolillo DJ Intracoronary brachytherapy after stenting *de novo* lesions in diabetic patients: results of a randomized intravascular ultrasound study. J Am Coll Cardiol. (2004) 44(3):520–7. 10.1016/j.jacc.2004.02.061 15358014

[18] Waksman R. Late thrombosis after radiation: sitting on a time bomb. Lippincott Williams & Wilkins. (1999) 100(8):780–2. 10.1161/01.CIR.100.8.780 10458709

[19] De Filippo O Wańha W Sanavia T Januszek R Giacobbe F Campo G Treatment of in-stent restenosis with ultrathin-strut versus thin-strut drug-eluting stents or drug-eluting balloons: a multicentre registry. EuroIntervention. (2024) 20(21):e1340. 10.4244/EIJ-D-24-00491 39492702 PMC11525456

