# Supplementary figures and images for: Intravascular brachytherapy vs. drug-coated balloons for in-stent restenosis in patients with diabetes
Source: Front Cardiovasc Med. 2026 Jan 12;12:1634096. doi: 10.3389/fcvm.2025.1634096 (PMC12832873; doi:10.3389/fcvm.2025.1634096)

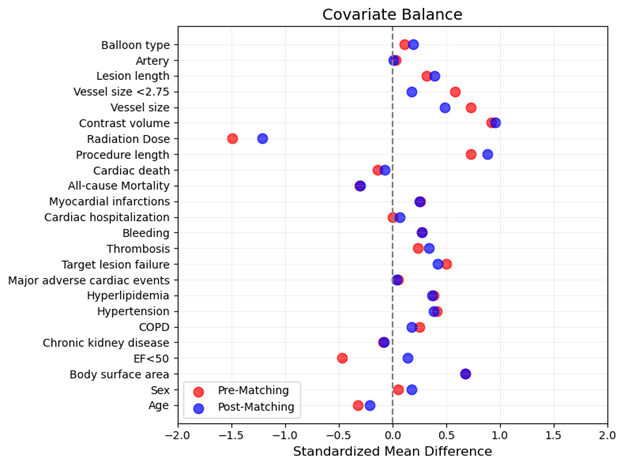

Supplement: Supplementary Figure A1 — Balance diagnostic pre- and post- matching by characteristics. [file Image1.png]
